# A framework: make it useful to guide and improve practice of clinical trial design in smaller populations

**DOI:** 10.1186/s12916-016-0752-x

**Published:** 2016-11-25

**Authors:** Kit C. B. Roes

**Affiliations:** Julius Centre for Health Sciences and Primary Care, Biostatistics and Research Support, University Medical Center Utrecht, Utrecht, The Netherlands

**Keywords:** Randomised clinical trials, Clinical trial design, Protocol development, Rare diseases

## Abstract

The increased attention to design and analysis of randomised clinical trials in small populations has triggered thinking regarding the most appropriate design methods for a particular clinical research question. Decision schemes and algorithms have been proposed, with varying starting points and foci. Parmar et al. (BMC Medicine 14:183, 2016) proposed a framework designed to assist the clinical trial team in design choices during protocol preparation. Herein, further stimulus is given regarding the extent to which a framework may help change practice for the better, the careful considerations for changing the usual error levels applied and the room for innovation in clinical trial design.

Please see related article: http://bmcmedicine.biomedcentral.com/articles/10.1186/s12916-016-0722-3.

## Background

The increased attention to design and analysis of randomised clinical trials in small populations has triggered thinking regarding the processes leading to the most appropriate design for a particular clinical research question. In common diseases, this might not seem a pressing problem, given the extensive practice- and theory-based experience in trial designs. In the context of drug development for rare diseases, guidance from the European Medicines Agency [1] states that “*in conditions with small and very small populations, less conventional and/or less commonly seen methodological approaches may be acceptable if they help to improve the interpretability of the study results*”. However, this advice does not provide practical guidance on how such choices can be made at the clinical trial designing stage. Moreover, it also states that “[n]*o methods exist that are relevant to small studies that are not also applicable to large studies*” [1]. Hence, such practical guidance is actually relevant for all trials. Thus, there is arguably a need for a design framework, with that proposed by Parmar, Sydes and Morris [2] having particularly strong points. Their framework follows a logical order of the steps one would take in designing a trial, allowing for practical implementation. Secondly, it is driven by what could be termed a ‘step-down approach’: at each step following a non-feasible option, the next potential change or comprise to be considered is the one with minimal impact on the objective of obtaining high quality randomised evidence to improve care for the target patient population. It also appropriately addresses the fact that designing a clinical trial is a complex multidisciplinary and multifaceted exercise, not easily captured in a simple decision scheme.

Frameworks (or even algorithms) for applying particular designs for randomised clinical trials in small populations have been previously proposed, notably by Gupta et al. [3] and Cornu et al. [4], both of which are based on a literature search up to 2010, a particular choice of decision ‘nodes’ and considerations of the pros and cons of (less familiar) designs. The decision nodes are driven by the type of intervention [3], type of outcome versus recruitment time [3, 4], feasibility of sample size [3], prior knowledge and treatment alternatives [3], and certain design desirabilities [4]. Further, they address minimising time on placebo, and/or ensuring that all participants are on active treatment at the end of the trial [4]. In these frameworks, it is actually difficult to ascertain whether a particular choice is (in some sense) the best possible given the circumstances. Indeed, the focus of Parmar et al.’s [2] framework on the ‘best’ randomised evidence to improve care for patients makes the search for this ‘best possible’ far more explicit. We concur that this requires the time and adequate attention of the entire clinical research team. If application of a framework helps to concentrate the team in order to design the best possible trial, this is, in itself, a positive effect not to be underestimated, particularly for investigator-initiated trials.

Herein, the proposed framework and its application are further considered, focusing on the level of ambition for its practical application, a discussion on relaxing type I or II errors, and the methods through which a deeper understanding of novel trial designs may be obtained.

## Frameworks as a starting point or to change practice for the better

In several instances, Parmar et al. [2] refer to standard practice as ‘traditional’ with an undoubtedly positive connotation. Rightfully so, many current practices in trial design are thoroughly founded on theory as well as extensive practical application. However, that does not hold for all framework aspects, and not all aspects improve clinical trial statistical efficiency. It is important to consider how the framework is best positioned for application. If it aims to stay as close possible to current practice (‘traditional’) to stimulate its use, the proposed ordering is acceptable. However, in clinical trial methodology there are a number of current practices that we (as statisticians) know are not optimal, but which are difficult to change in real life. One could argue that application of a framework is an opportunity to influence less optimal practices. Therefore, this could lead to some changes within the framework.

Two approaches labelled as ‘less common’ are (1) including covariate information and (2) moving from two- to one-sided significance tests; this really seems a missed opportunity to influence practice. Regarding the inclusion of covariate information, it is (by now) well accepted that including relevant prognostic covariates into the primary analysis will most likely increase power, and should be considered for any trial at the initial design stage. However, it remains at the ‘recommendation for improvement’ stage in small population trials [5]. On the same note, stratification of the randomisation is usually considered for every trial. For small populations, there are clear limits to the amount of ‘traditional’ stratification that can be performed. Both stratification and inclusion of covariate information should therefore be considered in concert for any trial at an early stage [6], which can be carried out fairly independently of the other trial features.

It is beyond the scope of this commentary (and maybe also beyond the author’s competence) to fully cover the discussion on one- or two-sided testing. What can be noted, however, is that group sequential and adaptive clinical trials can only be appropriately designed and understood with one-sided testing [7]. Given the widespread use of these flexible designs it could be concluded that this debate has effectively ended, as long as the ‘standard’ one-sided α-level is 2.5%. Other choices (ie moving to 5% one-sided) would then fall under a relaxation of the α-level.

## Relaxing power, relaxing the α-level

Parmar et al. [2] provide a thoughtful discussion on carefully relaxing the power or α, which is a particularly strong point in their framework. Relaxing the power and α-level strongly relates to the (theoretical) reproducibility of the trial. Society expects that research results are reliable, with even greater pressure when vulnerable patients have contributed to the research. Expressed concerns on reliability of research in general, and medical research in particular, have raised awareness on the rigor of design that is required [8]. One of the areas for improvement indicated is striking the right balance of clinically relevant outcomes, power and α-level. A careful approach, particularly in small populations, seems to be on the right direction.

## Deeper understanding of novel trial designs

Novel design and analysis approaches for small populations are expected to be beneficial in clinical research aimed at improving care for patients [9, 10]. A deeper understanding of the properties of such designs needs research as well as lessons learned from actual application. Our current deep level of understanding of the ins and outs of clinical trial design has only been reached because of a continuous cycle of improvement between theory development and real-life application. Hence, there is long-term benefit – beyond the level of an individual trial – of ‘road testing’ novel design features that hold strong promise of improvement based on theory. It will be worthwhile to consider whether application of a framework, as proposed, retains sufficient opportunity to experiment with trial design.
